# Associations between vaping and relapse to smoking: preliminary findings from a longitudinal survey in the UK

**DOI:** 10.1186/s12954-019-0344-0

**Published:** 2019-12-30

**Authors:** Leonie S. Brose, Julia Bowen, Ann McNeill, Timea R. Partos

**Affiliations:** Institute of Psychiatry, Psychology and Neuroscience, King’s College London, London, UK

## Abstract

**Background:**

Most smokers attempting to quit relapse. There is little evidence whether the use of e-cigarettes (‘vaping’) increases or decreases relapse. This study aimed to assess 1) whether vaping predicted relapse among ex-smokers, and 2) among ex-smokers who vaped, whether vaping characteristics predicted relapse.

**Methods:**

Longitudinal web-based survey of smokers, recent ex-smokers and vapers in the UK, baseline in May/June 2016 (*n* = 3334), follow-up in September 2017 (*n* = 1720). Those abstinent from smoking ≥ 2 months at baseline and followed up were included. Aim 1: Relapse during follow-up was regressed onto baseline vaping status, age, gender, income, nicotine replacement therapy use and time quit smoking (*n* = 374). Aim 2: Relapse was regressed onto baseline vaping frequency, device type, nicotine strength and time quit smoking (*n* = 159).

**Results:**

Overall, 39.6% relapsed. Compared with never use (35.9%), past/ever (45.9%; _adj_OR = 1.13; 95% CI, 0.61–2.07) and daily vaping (34.5%; _adj_OR = 0.61; 95% CI, 0.61–1.89) had similar odds of relapse, for non-daily vaping evidence of increased relapse was inconclusive (65.0%; _adj_OR = 2.45; 95% CI, 0.85–7.08). Among vapers, non-daily vaping was associated with higher relapse than daily vaping (_adj_OR = 3.88; 95% CI, 1.10–13.62). Compared with modular devices (18.9% relapse), tank models (45.6%; _adj_OR = 3.63; 95% CI, 1.33–9.95) were associated with increased relapse; evidence was unclear for disposable/cartridge refillable devices (41.9%; _adj_OR = 2.83; 95% CI, 0.90–8.95). Nicotine strength had no clear association with relapse.

**Conclusion:**

Relapse to smoking is likely to be more common among ex-smokers vaping infrequently or using less advanced devices. Research into the effects of vaping on relapse needs to consider vaping characteristics.

## Introduction

Smoking remains the primary preventable cause of illness and premature death in countries with a high socio-demographic index such as the United Kingdom (UK) [1]. Population surveys from the USA, UK, Canada and Australia show that at least a third of smokers have made a serious attempt to quit smoking in the past year [2, 3].

However, the vast majority of attempts are not successful. In unaided attempts, less than 5% are still abstinent 1 year after they made a quit attempt [4]. A recent review and modelling study showed that after 12 weeks of licenced pharmacotherapy, abstinence rates at 1 year were 23% for varenicline, the most effective treatment, 17% for bupropion, 13% for nicotine replacement therapy (NRT) and 8% for placebo. Abstinence rates drop most steeply in the first week, and after about a month, the rate of returning to smoking slows down considerably [5]. A return to smoking after an initial period of abstinence is generally defined as relapse, but there is no agreed definition of the length of the period of abstinence [6]. Interventions could reduce relapse by preventing initial brief lapses, preventing lapses from leading to full relapse or both [6]. However, there is little evidence on effective interventions to reduce relapse with evidence of a benefit available only for varenicline [6].

In addition to the licenced pharmacotherapies, e-cigarettes have become available as a quitting aid and since 2013, e-cigarette use (vaping) has been the most common form of support in attempts to quit smoking in England [7, 8]. Longitudinal studies assessing vaping and smoking cessation indicate that frequency of use and type of device used are important; daily use, in particular daily use of more advanced devices, has been associated with quitting behaviour and abstinence from smoking [9–11].

While many studies have looked at vaping and its effects on smoking cessation [8], to date, very little evidence is available on its effect on relapse. A recent analysis of a longitudinal US survey [12] found that former smokers who vaped daily or non-daily had a greater risk of relapse compared with never vapers. However, there was no minimum length of abstinence to be categorised as a former smoker and the study did not adjust for any vaping characteristics. A second study using the same survey separated out smokers who had quit for less or more than 12 months and assessed relapse associated with different frequencies of vaping [13]. In recent ex-smokers, vaping was not significantly associated with relapse at follow-up; in longer-term ex-smokers, prior vaping and current regular vaping were associated with higher relapse [13]. A recent qualitative study in the UK concluded that smoking lapses were perceived differently when e-cigarettes were used, with lapses seen as permissible [14]. The authors’ analysis of the experience of vapers quitting smoking suggests that vaping could support long-term relapse prevention. This is also supported by a recent randomised controlled trial showing higher abstinence and higher usage for e-cigarettes than for nicotine replacement therapy at 1-year follow-up [15].

In the UK, vaping has been increasing among longer-term ex-smokers [16, 17] and many vapers use e-cigarettes for a prolonged period of time, with one-third of current users in Great Britain having vaped for more than 2 years [8]. If vaping protected against relapse, increased use and longer-term use among ex-smokers would have a positive effect on public health. If however vaping among ex-smokers increased the risk of relapse, the increased uptake and prolonged use would have an overall negative effect.

The aims of this study were to assess in a sample of ex-smokers whether:

1. Vaping was associated with subsequent relapse to smoking when adjusting for demographics, current use of other nicotine and time since they quit smoking.
2. Among those who vaped, characteristics of vaping (frequency, device type, nicotine strength) were associated with subsequent relapse to smoking.

## Methods

### Study design and sample

This study used data from a longitudinal web-based survey of a national general population sample of smokers, ex-smokers and vapers aged 18 and over in the UK. Participants were recruited through Ipsos MORI, a leading market research organisation in the UK, from members of an online panel managed by Ipsos Interactive Services. At recruitment, quotas were imposed on demographics to include a representative sample of age, sex and geographical region. Panel members were invited via email to take part in the survey. To reduce bias, the email did not specify the topic of the survey. Panel members received points for completing surveys which were redeemable for shopping vouchers. Participants were asked to give consent electronically prior to commencing the survey and were screened for smoking status with only smokers and ex-smokers being eligible to take part. The questionnaires took an estimated 15 to 20 min to complete.

**Fig. 1:**
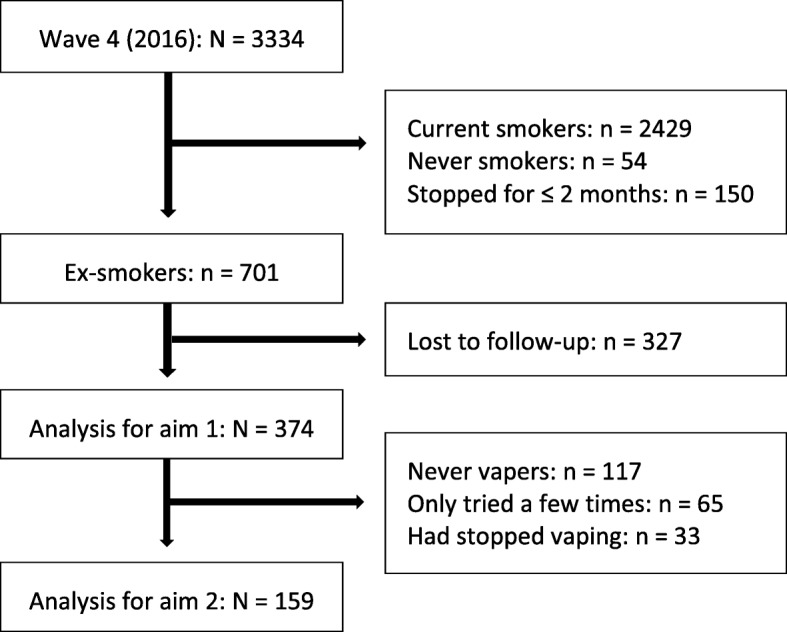
Flowchart of inclusion and exclusion of participants

There have been five waves of the survey: wave 1 in November/December 2012 (*N* = 5000), wave 2 in December 2013 (follow-up, *N* = 2182), and wave 3 in December 2014 (follow-up, *N* = 1519). At wave 1, all participants were smokers or recent ex-smokers, i.e. had smoked within the previous 12 months. Wave 4 was conducted in May/June 2016 (*N* = 3334); 933 participants were followed up from the previous three waves and an additional 2403 smokers, recent ex-smokers or exclusive vapers recruited. Wave 5 was completed in September 2017 (*N* = 1720) with 602 who were involved in all waves and the remainder followed up from wave 4. The present analyses only focused on data from wave 4 in 2016 and wave 5 in 2017; additional details about these are provided elsewhere [18, 19]. For both aims, only those who had quit smoking for more than 2 months at wave 4 and were successfully followed up at wave 5 were included in the analysis (*n* = 374, Fig. 1). For aim 2, the sample was further restricted to ex-smokers who vaped at wave 4 (*n* = 159, Fig. 1).

### Measures

All variables included in the analyses were collected at wave 4 except for the outcome variable which used data from wave 5.

Socio-demographic information included age in years (continuous). Gender was recorded as male and female. For annual income, respondents selected one response option (under £6500, £6500–£15,000, £15,001–£30,000, £30,001–£40,000, £40,001–£50,000, £50,001–65,000, £65,001–£95,000, £95,001 and over, ‘don’t know/prefer not to say’). The UK government defined ‘low income’ as below 60% of the national median, which in 2016/2017 equated to about £15,400 [20]. Therefore, responses were collapsed into ‘up to £15,000’ (low income), ‘£15,001 to £30,000’ (middle income) and ‘over £30,000’ (high income). Those who selected ‘don’t know/prefer not to say’ were retained as ‘not disclosed’. Ethnicity was recorded using UK census categories [21]. It was not included in the analysis because white English/Welsh/Scottish/Northern Irish/British was selected by 90.9% (*n* = 340) and all other categories by fewer than 10 participants (Additional file 1: Table S2). To avoid small groups, categories would have to be collapsed so much as to have little informative value.

Smoking status, time since quit smoking, vaping characteristics and NRT use were assessed using questions and response options detailed in Table 1.

**Table 1:** Measures related to smoking, vaping and nicotine replacement therapy use

Smoking status Could you please tell us which of the following best applies to you now? a) I smoke cigarettes (including hand-rolled) every day b) I smoke cigarettes (including hand-rolled), but not every day c) I do not smoke cigarettes at all, but I do smoke tobacco of some kind (e.g. pipe or cigar) d) I have stopped smoking completely in the last year (i.e. since May 2015)/since the last survey in December 2014) [In Wave 4, different time spans for previous participants and new recruits; in Wave 5, May/June 2016 for all participants] e) I stopped smoking completely more than a year ago (i.e. before May 2015)/before the last survey in December 2014 [In Wave 4, different time spans for previous participants and new recruits; in Wave 5, May/June 2016 for all participants] f) I have never been a smoker Categorised as current smoker (a, b, c); ex-smoker (d, e); excluded from survey (f)	
Time quit smoking How long ago did your most recent quit attempt start? By most recent, we mean the last time you tried to quit smoking a) In the last week b) More than a week and up to a month c) More than 1 month and up to 2 months d) More than 2 months and up to 3 months e) More than 3 months and up to 6 months f) More than 6 months and up to a year g) More than a year and up to 15 months h) Don’t know / can’t remember Ex-smokers categorised as 2–12 months (d–f); > 12 months (g); excluded (a–c)	
Vaping status Could you please tell us which of the following best applies to you now? a) I currently vape/use e-cigarettes daily b) I currently vape/use e-cigarettes but not every day c) I have tried vaping/an e-cigarette once or a few times d) I stopped vaping/using e-cigarettes in the last year [In wave 5, since May/June 2016] e) I stopped vaping/using e-cigarettes over a year ago [In wave 5, before May/June 2016] f) I have never vaped/used e-cigarettes Categorised as daily use (a); non-daily use (b); past/ever use (c-e); never vaped (f)	
Device type What electronic cigarette or vaping device do you currently use / did you use the most? a) A disposable e-cigarette or vaping device (non-rechargeable) b) An e-cigarette or vaping device that uses replaceable pre-filled cartridges (rechargeable) c) An e-cigarette or vaping device with a tank that you refill with liquids (rechargeable) d) A modular system that you refill with liquids (you use your own combination of separate devices: batteries, atomizers etc...) e) Don’t know Categorised as disposable, refillable with cartridges, don’t know (a, b, e); tanks (c); modular (d)	
Nicotine strength What strengths of nicotine do you use when vaping/using your e-cigarette? a) No nicotine b) 1 -8 mg/ml c) 9-14 mg/ml d) 15-20 mg/ml e) 21-24 mg/ml f) 25 mg/ml g) Don’t know Participants selecting multiple answers (*n* = 12) were asked “Which of these nicotine strengths do you use most often? Please select one” with the same responses as the initial question. Responses from both questions were combined to find the strength used/used most often. For main analysis, categorised as none or unknown (a, g); 1 to 14 mg/ml (b, c); 15 mg/ml and over (d–f). For sensitivity analysis, categorised as no nicotine (a); 1 to 8 mg/ml (b); 9 to 14 mg/ml (c); 15 mg/ml and over (d–f); g excluded.	
Nicotine replacement therapy (NRT) use Participants were asked three questions to determine NRT use 1. Which, if any, of the following are you currently trying to help you cut down the amount you smoke? 2. Do you regularly use any of the following in situations where you are not allowed to smoke? 3. Can I check, are you using any of the following for any reason at all? a) Nicotine gum b) Nicotine replacement lozenge/tablet c) nicotine replacement inhaler/inhalator d) Nicotine replacement nasal spray e) nicotine patch f) Electronic cigarette or vaping device ^1^ g) Nicotine mouthspray h) Another nicotine product i) Other, please type in [text box] j) None of these/not using anything k) Don’t know ^1^Not asked in question 3 Categorised as current NRT use (a–e, g in any of the three questions)	

The outcome variable was a binary categorisation of relapse to smoking, derived from smoking status at waves 4 and 5. Those identified as ex-smokers at wave 4 who gave a response at wave 5 other than ‘I stopped smoking completely before the last survey [wave 4]’ were categorised as having relapsed. This definition of relapse included those who were abstinent at follow-up but had lapses or relapsed to smoking in between waves.

### Analysis

Attrition analysis was conducted for all ex-smokers at wave 4; chi-square statistics were used to compare follow-up rates by time quit smoking, vaping status, gender, income and NRT use. Mean age for those followed up and lost to follow-up was compared using an independent groups *t* test.

- Aim 1: Relapse rates were calculated overall and by respondent characteristic. Bivariate and multivariable logistic regression was used to assess the association between relapse, vaping status (daily use, non-daily use, past/ever use compared with never use), gender, age, income, NRT use and time quit smoking.
- Aim 2: Relapse rates were calculated in this sample overall and by respondent characteristics. Bivariate and multivariable logistic regression was used to assess the association between relapse, frequency of e-cigarette use (non-daily compared with daily use), type of electronic cigarette used most, nicotine strength used most and time quit smoking. Bivariate analyses were also conducted to assess the associations between gender, age, income and NRT use, but because of the small sample size, the multivariable analysis did not adjust for these variables. Post-hoc sensitivity analyses were run without those (*n* = 15) who did not know the strength of nicotine they used and using four categories (no nicotine, 1 to 8 mg/ml, 9 to 14 mg/ml, 15 mg/ml and over).

In addition to the logistic regressions, Bayes factors were calculated for the association between vaping status and relapse to smoking for both aims. Bayes factors are the ratio of the (average) likelihood of two hypotheses being correct given a set of data. The two hypotheses are typically that an intervention had a desired effect (‘H_1_ hypothesis’) versus that it had no effect (‘null hypothesis’) [22]. Bayes factors are particularly useful for the evaluation of non-significant effects as they can indicate if there is support for either of the hypotheses or if the data are insensitive (e.g. due to small sample sizes). A Bayes factor of more than 3 can be taken as evidence against the null hypothesis and anything below 0.3 can be considered evidence for the null hypothesis. A Bayes factor between 0.3 and 3 is considered to indicate insensitive data [22]. This means that findings with *p* values greater than the commonly used 0.05 cut-off should only be presented as ‘lack of association’ if the Bayes factor is < 0.3 [22]. Calculation of Bayes factors requires specification of an expected effect size based on previous research. For the present calculation, the expected effect size was based on past research looking at relapse for other NRT users [23]. Using Dienes’ Bayes factor calculator [24], the mean was set at 0, the tails set to 2 and the logarithm of the odds ratio used for the sample mean.

## Results

### Attrition and sample characteristics

While follow-up rates were low from wave 4 to wave 5 (51.6%), follow-up for ex-smokers at wave 4 showed very little variation by time since they had quit smoking, vaping status, NRT use, gender or income; only age differed between groups with those followed up on average older than those lost to follow-up (Additional file 1: Table S1 for details).

Sample characteristics are presented for the two analyses in Table 2. Of all ex-smokers, 37.4% had stopped between 2 and 12 months before wave 4 and 62.6% over 12 months before wave 4; 42.5% were vaping, 26.2% had only tried a few times or had stopped vaping and 31.3% had never tried vaping at wave 4. In both samples (all ex-smokers and vaping ex-smokers), there were slightly more men than women, over two-thirds were aged between 40 and 54, around four in ten had a high income and just over 1 in 10 were using NRT. Among those who vaped, most vaped daily and about half used tank models; 11.3% used no nicotine, 9.4% did not know the nicotine content, 45.9% used 1 to 14 mg/ml and 33.3% used nicotine concentrations above 15 mg/ml (Table 2).

**Table 2:** Sample characteristics at wave 4 for the two analyses

		Ex-smokers, *n* = 374		Vaping ex-smokers, *n* = 159	
Age (years)	Mean (SD)	48.1 (15.3)		49.2 (14.1)	
		*n*	%	*n*	%
Gender	Female	171	45.7	70	44.0
	Male	203	54.3	89	56.0
Annual income	Low	62	16.6	27	15.1
	Moderate	107	28.6	46	28.9
	High	158	42.2	62	39.0
	Not disclosed	47	12.6	24	15.1
Vaping Status	Daily use	139	37.2	139	87.4
	Non-daily use	20	5.3	20	12.6
	Only tried a few times	65	17.4	-	-
	Stopped ≤ 1 year ago	20	5.3	-	-
	Stopped > 1 year ago	13	3.5	-	-
	Never vaped	117	31.3	-	-
Time quit smoking	2 to 12 months	140	37.4	50	31.4
	> 12 months	234	62.6	109	68.6
NRT use	No	326	87.2	141	88.7
	Yes	48	12.8	18	11.3
Device type	Disposable	-	-	5	3.1
	Refillable with cartridges	-	-	37	23.3
	Tank	-	-	79	49.7
	Modular	-	-	37	23.3
	Don’t know	-	-	1	0.6
Nicotine strength	No nicotine	-	-	18	11.3
	1 to 8 mg/ml	-	-	38	23.9
	9 to 14 mg/ml	-	-	35	22.0
	15 to 20 mg/ml	-	-	41	25.8
	21 to 24 mg/ml	-	-	9	5.7
	25 mg/ml and over	-	-	3	1.9
	Don’t know	-	-	15	9.4

*NRT* nicotine replacement therapy

### Aim 1: Association between vaping status and subsequent relapse to smoking

Overall, 39.6% of ex-smokers who had stopped for at least 2 months at wave 4 relapsed to smoking during the follow-up period. Compared with those who had never vaped (35.9% relapse), those who vaped non-daily had higher rates of relapse (65.0%, Bayes factor 1.01), although this association was weakened and no longer significant when adjusting for covariates (adjusted OR = 2.45; 95% CI, 0.85 to 7.08; *p* = 0.098; Table 3). Past/ever users (45.9%; Bayes factor 0.99; adjusted OR = 1.13; 95% CI, 0.61 to 2.07; *p* = 0.070) and daily users (34.5%; Bayes factor 0.99; adjusted OR = 1.07; 95% CI, 0.61 to 1.89; *p* = 0.80; Table 3) had relapse rates closer to those of never users. Time quit smoking was strongly associated with relapse with more recent ex-smokers more likely to relapse. Higher age was associated with reduced relapse. In unadjusted results, those using NRT were more likely to relapse. There was little difference by gender or income (Table 3). Relapse rates for respondent characteristics for all response options individually (where these were collapsed in the regressions) are provided in Additional file 1: Table S3.

**Table 3:** Aim 1: Association between wave 4 vaping status, socio-demographics, use of NRT and relapse to smoking during follow-up, *n* = 374

Wave 4 characteristic		% relapsed	Unadjusted (bivariate) analysis				Adjusted (multivariable) analysis			
			OR	95% CI		*p* value	OR	95% CI		*p* value
Vaping status	Daily use	34.5	0.94	0.56	1.58	0.82	1.07	0.61	1.89	0.80
	Non-daily use	65.0	3.32	1.23	8.96	0.018	2.45	0.85	7.08	0.098
	Past/ever use	45.9	1.52	0.88	2.62	0.14	1.13	0.61	2.07	0.70
	Never (ref)	35.9	1	1	1	Ref	1	1	1	Ref
Gender	Male	38.9	0.94	0.62	1.43	0.80	0.94	0.60	1.49	0.80
	Female (ref)	40.4	1	1	1	Ref	1	1	1	Ref
Age	Per year increase	-	0.97	0.96	0.98	< 0.001	0.98	0.96	0.99	0.002
Income	Not disclosed, low, moderate	36.6	0.74	0.49	1.13	0.17	0.87	0.55	1.39	0.31
	High (ref)	43.7	1	1	1	Ref	1	1	1	Ref
NRT use	Yes	43.7	1.98	1.07	3.64	0.029	1.42	0.72	2.78	0.31
	No (ref)	36.6	1	1	1	Ref	1	1	1	Ref
Time quit smoking	2 to 12 months	61.4	4.42	2.82	6.91	< 0.001	3.95	2.48	6.29	< 0.001
	> 12 months (ref)	26.5	1	1	1	Ref	1	1	1	Ref

*CI* confidence interval, *NRT* nicotine replacement therapy; *OR*, odds ratio

### Aim 2: Association between vaping characteristics and subsequent relapse to smoking

Among the smaller sample of ex-smokers who were vapers at wave 4, 38.4% relapsed. Non-daily use was associated with higher rates of relapse than daily use (65.0% versus 34.5%; Bayes factor 1.01; adjusted OR = 3.88; 95% CI, 1.10 to 13.62; *p* = 0.035; Table 4). Compared with vapers using modular devices (18.9%), those using any other device had higher rates of relapse. For those using disposable or cartridge refillable devices, this association with higher relapse was attenuated when adjusting for other characteristics (41.9%; adjusted OR = 2.83; 95% CI, 0.90 to 8.95; *p* = 0.076; Table 4); for tank models, the association remained significant (45.6%; adjusted OR = 3.63; 95% CI, 1.33 to 9.95; *p* = 0.012; Table 4). Nicotine strength had no clear association with relapse (compared with 15 mg/ml and over: 1 to 14 mg/ml: adjusted OR = 1.51; 95% CI, 0.66 to 3.44; *p* = 0.33; no nicotine or unknown nicotine strength: adjusted OR = 0.54; 95% CI, 0.17 to 1.74; *p* = 0.30; Table 4). The post-hoc sensitivity analysis indicated little change in relapse rates for the remaining ‘no nicotine’ group (38.9% relapse versus 36.4% when combined with ‘unknown’) and similar relapse rates across the two categories that were combined in the main analysis (1 to 8 mg/ml, 44.7% relapse; 9 to 14 mg/ml, 45.7% relapse). As before, shorter time since quit smoking was strongly associated with relapse (Table 4). Relapse rates for respondent characteristics without collapsing response options are provided in Additional file 1: Table S3.

**Table 4:** Aim 2: Associations between wave 4 frequency of e-cigarette use, device type, nicotine strength, use of NRT, time quit smoking, socio-demographics and relapse to smoking during follow-up, *n* = 159

		% relapsed	Unadjusted (bivariate) analysis				Adjusted (multivariable) analysis			
			OR	95% CI		*p* value	OR	95% CI		*p* value
Vaping status	Non-daily use	65.0	3.52	1.32	9.41	0.012	3.88	1.10	13.62	0.035
	Daily use (ref)	34.5	1	1	1	Ref	1	1	1	Ref
Device type	Disposable, cartridges, unknown	41.9	3.09	1.11	8.57	0.031	2.83	0.90	8.95	0.076
	Tanks	45.6	3.59	1.41	9.13	0.007	3.63	1.33	9.95	0.012
	Modular (ref)	18.9	1	1	1	Ref	1	1	1	Ref
Nicotine strength	None, unknown	36.4	1.32	0.53	3.32	0.55	0.54	0.17	1.74	0.30
	1 to 14 mg/ml	45.2	1.91	0.91	4.02	0.090	1.51	0.66	3.44	0.33
	15 mg/ml and over (ref)	30.2	1	1	1	Ref	1	1	1	Ref
NRT use	Yes	50.0	1.71	0.64	4.58	0.29	1.25	0.41	3.80	0.70
	No (ref)	36.9	1	1	1	Ref	1	1	1	Ref
Time quit smoking	2 to 12 months	64.0	4.90	2.39	10.04	< 0.001	4.62	2.11	10.09	< 0.001
	> 12 months (ref)	26.6	1	1	1	Ref	1	1	1	Ref
Gender	Male	38.2	0.99	0.52	1.87	0.96				
	Female (ref)	38.6	1	1	1	Ref				
Age	Per year increase	41.7	0.98	0.96	1.00	0.068				
Income	Not disclosed, low, moderate	35.1	0.70	0.36	1.34	0.28				
	High (ref)	43.5	1	1	1	Ref				

*CI* confidence interval, *NRT* nicotine replacement therapy, *OR* odds ratio

## Discussion

In a group of ex-smokers who had stopped for at least 2 months, those who used e-cigarettes infrequently at baseline were somewhat more likely to relapse to smoking over the following 15-month period than those who had never used e-cigarettes, whereas those who used e-cigarettes daily were just as likely to relapse as never users. When just examining vapers at baseline, non-daily users were more likely to relapse than daily users. Those using devices other than modular devices also appeared to be more likely to relapse to smoking whereas there was little association between nicotine strength used and relapse.

Infrequent vaping was associated with higher relapse to smoking in vapers. Vaping and also use of NRT may be a marker of higher dependence on nicotine which is consistently associated with increased relapse [25]. This is supported by evidence that vapers were more likely than non-vapers to report higher cigarette dependence when they were smoking [26]. Infrequent use and thus nicotine consumption would not sufficiently protect against this increased risk of relapse. Moving from identifying as a smoker to identifying oneself as an ex-smoker is also important in maintaining long-term abstinence [27] and it could be speculated that infrequent vapers have not fully switched to a new identity [28]. Vaping can also increase exposure to smoking-related cues, particularly if vapers use designated cigarette smoking areas.

Nicotine strength was not associated with relapse; this may partly be because the amount of nicotine available to the user is not solely determined by the concentration of nicotine in the liquid but also by the amount of liquid consumed, user characteristics such as puffing patterns, and the device type and device settings used [29–32]. In addition, the small sample in this analysis made it difficult to find an effect, and the grouping of strengths may have masked smaller effects, although this was not apparent in the sensitivity analysis which used more precise groupings. We categorised according to the strength used most often but only a small group of users (*n* = 12) used more than one strength.

There are a few limitations that need to be considered when interpreting the findings. Firstly, there was a large attrition rate between waves 4 and 5. However, follow-up rates for ex-smokers and for ex-smokers who vaped were very similar to the overall sample suggesting that findings would have been similar with higher follow-up rates. The small sample sizes available in this study may have failed to detect statistically significant effects due to low power. It was not possible to include information on smoking dependence prior to quitting or motivation to remain quit from smoking, variables that affect the chances of abstinence. Categories for variables were broad and more fine-grained information on vaping characteristics may have provided further insight.

The calculated Bayes factors for vaping were close to 1 for all categories, indicating that the data are insensitive rather than evidence for a lack of association [33]. As there was a lack of previous research looking at e-cigarettes and relapse, the effect size for the calculation was based on past research looking at relapse for NRT users [23] and may not directly apply to this research. All data were self-reported, which may result in recall or social desirability bias and did not allow for biochemical verification of abstinence. However, previous evidence indicates that levels of misreporting of abstinence in surveys are low [34]. There are likely to be additional confounding factors not accounted for in the present study which may affect the association between characteristics and relapse. For example, other studies found that vapers had been more dependent smokers [26] and that in general, late relapse to smoking was predicted by self-efficacy and dependence [35, 36], factors we were unable to include in this analysis. Finally, as this is an observational study, we cannot assign causality to the observed associations.

A strength of this study is that the sample came from a general population survey. It is also one of the first studies to look at vaping and relapse to smoking and the first to explore associations between device type and nicotine content. It is worth noting that this analysis included ex-smokers regardless of the type of support they had initially used when quitting smoking.

Findings for relapse in the present analysis are in line with previous research for smoking cessation showing differences in the likelihood of quitting smoking by type of device and frequency of use; where compared with non-e-cigarette users, non-daily cigalike (disposable/pre-filled) users were less likely to quit [11]. More advanced devices are likely to be more efficient at delivering nicotine and offer a longer battery life [37]. Notwithstanding the link between nicotine delivery and abstinence, qualitative evidence suggests individuals may create a bespoke successful approach to suit their needs [28]. Analyses of US survey data from 2013 to 2015 found that non-daily e-cigarette users had a greater risk of smoking relapse compared with never users [12], which, when separating out recent and longer-term ex-smokers, remained true for non-regular use among recent ex-smokers [13]. However, unlike the present study, those analyses also found increased relapse for daily use overall and for regular use among longer-term ex-smokers. The group of longer-term ex-smokers likely included former smokers of decades whereas in the present study, no one had stopped before 2012 and most had stopped within the past two years, thus making the sample more similar to the recent ex-smokers in the US data. The years of data collection are likely to be relevant for associations between vaping and smoking behaviour as devices are evolving constantly. Additionally, vaping and smoking statuses were assessed differently in the different surveys and the present study used a stricter criterion of relapse. Similar to findings from smoking cessation [38–40], older participants were more likely to remain abstinent from smoking.

Future research looking at associations between vaping and relapse to smoking needs to include information on the duration of cessation, prior smoking characteristics (such as dependence) and more detailed information on characteristics of vaping such as frequency (ideally beyond a binary measure of daily/non-daily), device type and nicotine content. Interactions between these variables should be considered; there are for example likely to be interactions between type of device and frequency of use as well as between type of device and nicotine strength. The role of dependence and motivation to remain abstinent from smoking should be examined in sufficiently large samples.

## Conclusion

In a group of ex-smokers who had stopped smoking for at least 2 months, relapse to smoking during a 15-month follow-up period was likely to be more common among those who at baseline vaped infrequently or used less advanced devices. Research into the effects of vaping on relapse to smoking needs to consider characteristics of use including devices used and frequency of use.

## Supplementary information

**Additional file 1: Table S1.** Attrition rates for all wave 4 ex-smokers. **Table S2.** Ethnicity, n=374. **Table S3.** Relapse by respondent characteristic showing all responses individually for variables where response options were combined for the main analysis.

## Data Availability

The datasets analysed during the current study are available from the corresponding author on reasonable request.

## Abbreviations

CI: Confidence interval
NRT: Nicotine replacement therapy
OR: Odds ratio
