# Supplementary material for: Associations between vaping and relapse to smoking: preliminary findings from a longitudinal survey in the UK
Source: Harm Reduct J. 2019 Dec 30;16:76. doi: 10.1186/s12954-019-0344-0 (PMC6938007; doi:10.1186/s12954-019-0344-0)
Supplement: Supplementary file 1 — Additional file 1: Table S1. Attrition rates for all wave 4 ex-smokers. Table S2. Ethnicity, n=374. Table S3. Relapse by respondent characteristic showing all responses individually for variables where response options were combined for the main analysis. [file 12954_2019_344_MOESM1_ESM.docx]

**Associations between vaping and relapse to smoking – Preliminary findings from a longitudinal survey in the United Kingdom.
Leonie S Brose, Julia Bowen, Ann McNeill, Timea R Partos**

**Harm Reduction Journal**

**Supplementary material**

**Table S1. Attrition rates for all wave 4 ex-smokers.**

|  |  | **Followed up** | **Comparison** |
| --- | --- | --- | --- |
| Time quit smoking | 2 to 12 months | 51.1% | χ²(1)=1.0, p=0.33 |
|  | >12 months | 54.9% |  |
| Vaping status | Daily | 51.6% | χ²(3)=4.9, p=0.18 |
|  | Non-daily | 54.9% |  |
|  | Ever/past | 49.6% |  |
|  | Never | 58.8% |  |
| Gender | Male | 55.1% | χ²(1)=1.1, p=0.29 |
|  | Female | 51.5% |  |
| Income | Not disclosed, low, moderate | 52.4% |  |
|  | High | 62.8% |  |
| NRT use | Yes | 52.9% | χ²(1)=0.8, p=0.36 |
|  | No | 57.4% |  |
|  |  |  |  |
|  | **Not followed up** | **Followed up** |  |
| Age, Mean (standard deviation) | 42.2 (15.3) | 47.7 (15.3) | t=5.2, p<0.001 |

**Table S2. Ethnicity, n=374**

| **Ethnic group** | **n** | **%** |
| --- | --- | --- |
| White English / Welsh / Scottish / Northern Irish / British | 340 | 90.9 |
| Any other white | 12 | 3.2 |
| Mixed / multiple ethnic groups | 4 | 1.1 |
| Asian / Asian British | 11 | 2.9 |
| Black / African / Caribbean / Black British | 5 | 1.3 |
| Other ethnic group | 0 | 0 |
| Prefer not to say | 2 | 0.5 |

**Table S3. Relapse by respondent characteristic showing all responses individually for variables where response options were combined for the main analysis.**

| **Ex-smokers, n=374** |  | **n relapsed** | **% relapsed** |
| --- | --- | --- | --- |
| **Vaping Status** | Daily use | 48 | 34.5 |
|  | Non-daily use | 13 | 65.0 |
|  | Only tried a few times | 30 | 46.2 |
|  | Stopped ≤ 1 year ago | 10 | 50.0 |
|  | Stopped > 1 year ago | 5 | 38.5 |
|  | Never vaped | 42 | 35.9 |
| **Age (years)** | 18-24 | 16 | 57.1 |
|  | 25-39 | 37 | 45.1 |
|  | 40-54 | 63 | 46.0 |
|  | 55 and over | 32 | 25.2 |
| **Annual income** | Low | 16 | 35.5 |
|  | Moderate | 22 | 38.3 |
|  | High | 41 | 43.7 |
|  | Not disclosed | 69 | 34.0 |
| **Vaping ex-smokers, n=159** |  | **n relapsed** | **% relapsed** |
| **Device type used most** | Disposable | 2 | 40.0 |
|  | Refillable w cartridges | 16 | 43.2 |
|  | Tank | 36 | 45.6 |
|  | Modular | 7 | 18.9 |
|  | Don’t know | 0 | 0 |
| **Nicotine strength used most** | No nicotine | 7 | 33.3 |
|  | 1 to 8mg/ml | 17 | 44.7 |
|  | 9 to 14 mg/ml | 16 | 45.7 |
|  | 15 to 20 mg/ml | 11 | 26.8 |
|  | 21 to 24 mg/ml | 3 | 33.3 |
|  | 25 mg/ml and over | 2 | 66.7 |
|  | Don’t know | 5 | 41.7 |
